# GAPDH Released from *Lactobacillus johnsonii* MG Enhances Barrier Function by Upregulating Genes Associated with Tight Junctions

**DOI:** 10.3390/microorganisms11061393

**Published:** 2023-05-25

**Authors:** Mengying Lyu, Yuying Bai, Kanami Orihara, Kazuhiko Miyanaga, Naoyuki Yamamoto

**Affiliations:** 1 School of Life Science and Technology, Tokyo Institute of Technology, Yokohama 226-8501, Japan; 2 Department of Infection and Immunity, School of Medicine, Jichi Medical University, 3311-1, Yakushiji, Tochigi 329-0489, Japan

**Keywords:** *Lactobacillus johnsonii*, tight junctions, GAPDH, JAM-2, Caco-2 cells

## Abstract

Extracellular glyceraldehyde-3-phosphate dehydrogenase (GAPDH) has multiple interactions with various gut epithelial components. For instance, GAPDH in *Lactobacillus johnsonii* MG cells interacts with junctional adhesion molecule-2 (JAM-2) in Caco-2 cells and enhances tight junctions. However, the specificity of GAPDH toward JAM-2 and its role in the tight junctions in Caco-2 cells remain unclear. In the present study, we assessed the effect of GAPDH on tight junction regeneration and explored the GAPDH peptide fragments required for interaction with JAM-2. GAPDH was specifically bound to JAM-2 and rescued H_2_O_2_-damaged tight junctions in Caco-2 cells, with various genes being upregulated in the tight junctions. To understand the specific amino acid sequence of GAPDH that interacts with JAM-2, peptides interacting with JAM-2 and *L. johnsonii* MG cells were purified using HPLC and predicted using TOF–MS analysis. Two peptides, namely ^11^GRIGRLAF^18^ at the N-terminus and ^323^SFTCQMVRTLLKFATL^338^ at the C-terminus, displayed good interactions and docking with JAM-2. In contrast, the long peptide ^52^DSTHGTFNHEVSATDDSIVVDGKKYRVYAEPQAQNIPW^89^ was predicted to bind to the bacterial cell surface. Overall, we revealed a novel role of GAPDH purified from *L. johnsonii* MG in promoting the regeneration of damaged tight junctions and identified the specific sequences of GAPDH involved in JAM-2 binding and MG cell interaction.

## 1. Introduction

The human gut microbiota plays a pivotal role in the maintenance of human health by colonising the gut and stimulating the immune system. Research conducted in the last 20 years has been important in understanding this host–microbe interaction. The commensal intestinal microbiota interacts with gut epithelial cells to survive in the host by binding to specific host receptors, such as mucin and fibronectin. Pattern recognition receptors (PRRs) on intestinal epithelial cells, which sense and communicate with intestinal bacteria, have been extensively studied [1,2]. The intestinal epithelium provides physical barriers to maintain the intestinal mucosa and prevent mucosal bacterial invasion. The crosstalk between commensal bacteria and the intestinal mucosa is mediated by the bacterial surface and epithelial mucus proteins [3,4,5,6,7]. *Limosilactobacillus reuteri* strains with C-type lectins exert immunoregulatory effects in the gut by interacting with dendritic cells [8]. MapA adheres to collagen and Caco-2 cells [9]. Notably, Roos et al. [10] studied the mucus-binding protein (MUB) in *L. reuteri* 1063.

In general, the complex interplay between bacterial moonlighting proteins and host receptors is considered to contribute to the successful colonisation of hosts and lead to different host immune responses [11,12,13], although bacterial moonlighting proteins have no anchor sequence at the C-terminus for bacterial cell surface interactions and no secretion signals at the N-terminus [14]. Glyceraldehyde-3-phosphate dehydrogenase (GAPDH) present on the surface of *Lactobacillus jensenii* [15] and *Lactiplantibacillus plantarum* [16,17] possesses adhesive functions. It also acts as an adhesion factor for host epithelial mucin [17] and fibronectin [18]. In our previous study, commensal intestinal bacteria interacting with mouse gut surface proteins were screened from faecal bacterial samples to identify specific bacterial species and key host molecules in order to understand the role of these interactions in maintaining host health. Among the screened intestinal lactic acid bacteria, the frequently isolated *L*. *johnsonii* MG was selected by focusing on commensal bacteria with an affinity for gut epithelial surface proteins [19]. *L*. *johnsonii* MG repaired the tight junctions of hydrogen peroxide (H_2_O_2_)-damaged Caco-2 cells through the interaction of GAPDH located on the cell surface with junctional adhesion molecule-2 (JAM-2) and the subsequent upregulation of genes associated with tight junctions, anti-inflammatory effects, transcriptional regulation, and apoptosis in Caco-2 cells. The role of GAPDH located on the cell surface of *L. johnsonii* MG in interactions with JAM-2 was obvious in the previous study. However, the impact of free GAPDH without cellular components on interactions with tight junctions, its role in repairing damaged tight junctions, and its specific interaction with JAM-2, among multiple other interactions, remain unclear.

In the present study, we evaluated the barrier repair function of purified GAPDH without cellular components in H_2_O_2_-damaged Caco-2 cells, compared with its functioning in *L. johnsonii* MG. We assessed the interaction of free GAPDH with JAM-2, and investigated essential peptide fragments of GAPDH for JAM-2 binding and *L*. *johnsonii* MG localisation, through high-performance liquid chromatography (HPLC), purification, and docking analysis; this was based on the interactions noted in the 3D structure of GAPDH and JAM-2. 

## 2. Materials and Methods

### 2.1. Reagents 

Cy3 dye was obtained from GE Healthcare (Little Chalfont, UK). Casein, bovine serum albumin (BSA), α-chymotrypsin, trifluoroacetic acid (TFA), and acetonitrile were purchased from Nacalai Tesque, Inc. (Kyoto, Japan). Dulbecco’s modified Eagle’s medium (DMEM) and Gibco penicillin–streptomycin (10,000 U/mL) were obtained from Thermo Fisher Scientific (Waltham, MA, USA). Foetal bovine serum (FBS) was obtained from Biowest (Nuaillé, France). H_2_O_2_ was purchased from FUJIFILM Wako Pure Chemical, Inc. (Osaka, Japan). Polyclonal rabbit anti-ZO-1, anti-JAM-2, and anti-GAPDH antibodies were obtained from GeneTex Inc. (Irvine, CA, USA). The Cy3-conjugated anti-rabbit IgG antibody was obtained from Novus Biologicals (Centennial, CO, USA).

### 2.2. Affinity Purification

*L. johnsonii* MG was cultured in 200 mL MRS culture medium (Becton, Dickinson and Company, East Rutherford, NJ, USA) for 24 h at 37 °C under anaerobic conditions. The cultured bacterial cells were harvested via centrifugation at 13,000× *g* for 10 min and washed twice with 10 mM phosphate-buffered saline (PBS; pH 7.4, 0.8% NaCl). Bacterial surface layer proteins (Slps), including GAPDH, were released by washing with 5 M LiCl and dialysed with a Visking tube (MWCO = 10,000) against PBS. The dialysed Slps were loaded onto an affinity column (diameter, 12 mm × 25 cm) to purify the GAPDH. To prepare the affinity resin, an anti-GAPDH antibody diluted with 0.1 M NaHCO_3_ (1/500) was mixed with Profinity Epoxide (Bio-Rad Inc., Hercules, CA, USA) pre-equilibrated with 0.1 M NaHCO_3_. The LiCl-released fraction was applied to the affinity column, and the GAPDH was eluted from the column using 3 M KSCN (pH 6.0) after washing with 10 mM PBS.

### 2.3. Binding of GAPDH to JAM-2

To examine the specific binding between GAPDH and JAM-2, an anti-JAM-2 antibody was immobilised on 96-well immuno plates (Thermo Scientific Nunc, Waltham, MA, USA) for 24 h at 4 °C. Caco-2 cells (RCB0988) were purchased from the Riken Cell Bank (Tsukuba, Japan) and maintained in a 24-well culture plate (Thermo Fisher Scientific, Tokyo, Japan) for 10 days, washed with PBS, and harvested. Subsequently, the cell surface components were extracted using 0.1% Triton–PBS (cell extract). The cell extract was centrifuged at 8000× *g* for 10 min to remove aggregates and cell debris. After centrifugation, the supernatant was added to the plate and incubated for 2 h at 25 °C. Thereafter, 1% (*w*/*v*) BSA–PBS was added to block the plate after washing with PBS. Purified GAPDH was labelled with Cy3 using the Cy3 Mono-Reactive Dye Pack (GE Healthcare Japan Corporation, Tokyo, Japan), according to the manufacturer’s instructions. Cy3-labelled GAPDH was incubated with JAM-2 captured by the anti-JAM-2 antibody for 2 h at 25 °C. After removing unbound Cy3-labelled GAPDH by washing with PBS, the binding activity was quantified using a microplate reader (Varioskan LUX SkanIt Software 4.0; Thermo Fisher Scientific). BSA–PBS was used as the control instead of the anti-JAM-2 antibody. 

### 2.4. Tight Junction Preparation and Transepithelial Electrical Resistance (TEER) Measurement

Caco-2 cell culture and cell monolayers were prepared according to the method described by Bai et al. [19]. Intestinal barrier integrity was determined by measuring the TEER across the Caco-2 cell monolayer using Millicell^®^ ERS-2 (EMD Millipore Corporation, MA, USA). Differentiated Caco-2 cells with a TEER above 550 Ω cm^2^ were treated with 150 μM H_2_O_2_–DMEM (without FBS) for 0.2 h to damage the tight junctions. The medium was then replaced with fresh medium. Changes in the TEER were measured at 0, 0.2, 1, 2, 3, 4, 5, 6, and 7 h after treatment with the proteins or bacterial cells (n = 3). 

### 2.5. Bacterial Binding to Caco-2 Cells

To confirm the involvement of GAPDH in tight junction binding, the tight junctions of Caco-2 cells were prepared by cultivation in DMEM containing 10% FBS, as described above. Caco-2 cells were fixed using the BD Cytofix/Cytoperm^TM^ Solution Kit (BD Biosciences, San Diego, CA, USA), permeabilised with 0.5% Triton–PBS (pH 7.4), incubated with a rabbit anti-ZO-1 antibody (GeneTex, Irvine, CA, USA) after dilution with 1% casein–PBS (1/1000), and stained with a secondary antibody (Cy3-conjugated anti-rabbit IgG). *L. johnsonii* MG cells were treated with 5 M LiCl to completely remove surface-bound proteins (−GAPDH). Thereafter, purified GAPDH was reassociated on the *L*. *johnsonii* MG cell surface (+GAPDH). *L. johnsonii* MG cells were labelled with 5-(and 6-)carboxyfluorescein diacetate succinimidyl ester (CFDA/SE) (Nacalai Tesque) via incubation at 37 °C for 1 h. Excess CFDA was removed by centrifugation [CFDA–MG (−GAPDH) and CFDA–MG (+GAPDH)]. CFDA–MG (−GAPDH) and CFDA–MG (+GAPDH) were incubated with Caco-2 cells stained with the anti-ZO-1 antibody. *L. johnsonii* MG cells bound to Caco-2 cells were observed using the ZOE^TM^ Fluorescent Cell Imager (Bio-Rad); the numbers of bacteria on tight junctions and not on tight junctions were then determined.

### 2.6. Gene Expression in Caco-2 Cells

Caco-2 cells were seeded at a density of 5 × 10^4^ cells/well in nine wells (three wells each for three groups) of a 24-well culture plate (Thermo Fisher Scientific, Japan). The medium was replaced every 3 days, and the Caco-2 monolayers were grown for more than 10 days to enable differentiation. All wells were treated with 150 μM H_2_O_2_–DMEM without FBS for 10 min, and the medium was replaced with fresh medium containing *L. johnsonii* MG cells (5 × 10^6^ CFU/mL; H_2_O_2_–MG group), GAPDH (50 μg; H_2_O_2_–GAPDH group), and DMEM (H_2_O_2_ group). After 6 h of cultivation, Caco-2 cells were harvested and the total RNA was extracted using the RNeasy Mini Kit (QIAGEN, Hilden, Germany). The RNA was then reverse-transcribed into complementary DNA (cDNA) using a quantitative polymerase chain reaction (qPCR) reverse transcription master mix, according to the manufacturer’s instructions. Tight-junction-related genes were selected based on our previous study [19] and specific primers were prepared to quantify gene expression in triplicate, as listed in Table S1. The data were normalised to the mRNA expression level of β-actin, a housekeeping gene. Three parallel experiments were conducted for each group.

### 2.7. Ex Vivo Adhesion Test

The animal experiment was approved by the Animal Experiment Committee at the Tokyo Institute of Technology (authorisation number: D2020019) and conducted in accordance with the guidelines. BALB/c mice (female, 15 weeks old, 23–25 g) were obtained from Charles River Laboratory, Japan. Three mice were euthanised after 3 h of fasting, and their intestines were removed. Freshly extracted intestines from the BALB/c mice were used to determine the effects of GAPDH on the binding of *L*. *johnsonii* MG to the mouse gut. After washing with ice-cold PBS, the intestinal samples were fixed in 10% (*v*/*v*) formalin in PBS for 30 min at room temperature. The bacterial cells were treated as described previously. The bacterial samples [CFDA–MG (−GAPDH) and CFDA–MG (+GAPDH)] were injected into fixed mouse intestines and incubated at 37 °C for 30 min. The recovery levels of the CFDA–MG (−GAPDH) and CFDA–MG (+GAPDH) samples (n = 5) were assessed using a plate reader (Varioskan LUX SkanIt Software 4.0; Thermo Fisher Scientific). 

### 2.8. Characterisation of the Region of GAPDH That Binds with MG Cells and JAM-2 Protein

To obtain the peptide fragments of GAPDH, 50 µg of purified GAPDH was incubated with 50 µg of chymotrypsin for 2 h at 37 °C. After removing Slps using 5 M LiCl for 2 h at 30 °C and collecting the supernatant, the resulting peptides were incubated with MG cells. GAPDH fragments that bound or did not bind to MG cells (−Slps) were analysed by reversed-phase HPLC (RP-HPLC) using an XBridge BEH C18 column (4.6 mm × 150 mm; Waters Company, Milford, MA, USA). Separation was achieved using a linear gradient from 0% to 100% solvent B in solvent A for 40 min (A: 0.1% TFA in H_2_O; B: 0.1% TFA in acetonitrile) at a flow rate of 1 mL/min. Peptides showing decreased peak heights in the HPLC analysis after the binding treatment were collected (bound peptides). To confirm which peptides interacted with JAM-2, the anti-JAM-2 antibody coated on a 96-well microplate (Violamo, China) was blocked with 1% BSA–PBS for 1 h at 25 °C. JAM-2 in the Caco-2 cell extract was then added to each well of the microplate and incubated for 2 h at 25 °C. The GAPDH-digested peptides were incubated with the JAM-2 that was captured on the microplate at 25 °C. After 2 h of incubation, the supernatant was collected and analysed by HPLC, as described above.

### 2.9. Sodium Dodecyl Sulphate–Polyacrylamide Gel Electrophoresis (SDS–PAGE) 

GAPDH purified using affinity chromatography was analysed by performing SDS–10% PAGE, according to the method of Laemmli [20]. The protein was mixed with a 0.2 volume of sample buffer (125 nM Tris–HCl, 4% sodium dodecyl sulfate, 20% glycerol, 0.012% bromophenol blue, and 10% 2-mercaptoethanol) and heated for 5 min at 95 °C. The protein bands were visualised by staining the gels with Coomassie brilliant blue (CBB). Protein Ladder One Plus (Nacalai Tesque, Kyoto, Japan) was used as a size marker.

### 2.10. Bioinformatics Analysis

The amino acid sequence of the GAPDH from *L. johnsonii* MG was predicted in a previous study [19]. A template search for GAPDH was performed using the SWISS-MODEL (https://swissmodel.expasy.org/ (accessed on 18 January 2023)). Templates with the highest-scoring crystal structures were selected for model building. The NetSurfP server (https://services.healthtech.dtu.dk/service.php?NetSurfP-2.0 (accessed on 18 January 2023)) was used to predict the surface accessibility, secondary structure, and disorder of GAPDH. Basic physical and chemical parameters were predicted using the Compute PI/Mw tool (https://web.expasy.org/compute_pi/ (accessed on 6 March 2023)).

### 2.11. Docking Analysis

Molecular docking simulations were performed using the CDOCKER [21] program in the Discovery Studio (DS) 2018 software. The latest version of AlphaFold (https://alphafold.ebi.ac.uk (accessed on 9 March 2023)) [22] was used to predict the 3D structure of the JAM-2 sequence. The CHARMM force field base docking tool was used in CDOCKER for performing molecular dynamics module calculations to predict the putative peptide–protein complexes. Interaction energies were calculated after docking.

### 2.12. Statistical Analysis

Statistical significance was assessed by one-way or two-way ANOVA, using GraphPad Prism software (version 9.1). Statistical significance was set at *p* < 0.05.

## 3. Results

### 3.1. Adherence of MG Cells to Tight Junctions

**Figure 1:**
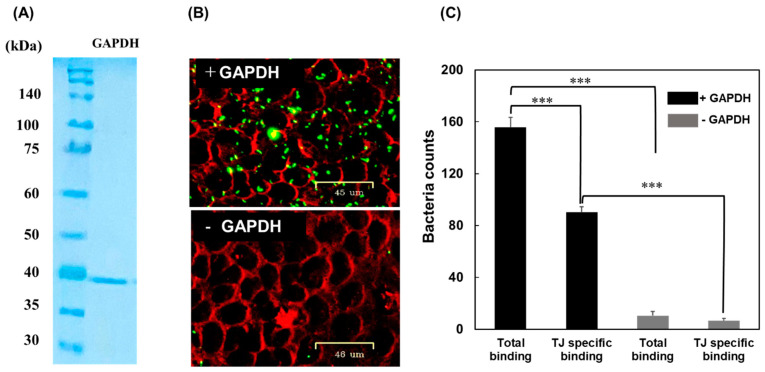
SDS–10% PAGE analysis of purified GAPDH using an anti-GAPDH antibody-coupled affinity resin (**A**). Binding of *L. johnsonii* MG to Caco-2 tight junctions, with or without GAPDH (**B**). CFDA-labelled *L. johnsonii* MG cells were incubated with Caco-2 cells stained with an anti-ZO-1 antibody and Cy3-conjugated anti-mouse IgG. Bacterial counts on tight junctions and total regions (**C**). Error bars represent the mean ± SEM (n = 6); *** *p* < 0.001 (one-way ANOVA).

Our previous study suggested that the binding of *L. johnsonii* MG to the JAM-2 protein is involved in the tight junction matrix in Caco-2 cells [19]. In the present study, the localisation of *L. johnsonii* MG in Caco-2 cells and the importance of GAPDH in this adherence were assessed. To explore the effect of GAPDH on the integrity of gut barrier function, GAPDH was purified via affinity chromatography. Slps released from *L. johnsonii* MG cells using 5 M LiCl were loaded onto an affinity resin coupled with an anti-GAPDH antibody. The GAPDH captured by specific antibodies was eluted from the affinity column using 3 M KSCN and 10 mM phosphate buffer (3 M KSCN–PB) after washing with 1 M NaCl–PB. After affinity purification, protein purity was confirmed by SDS–10% PAGE through CBB staining. A dense band with a molecular weight of approximately 40 kDa corresponding to GAPDH was observed in this fraction (Figure 1A).

The predominantly used Caco-2 cells, which are derived from human colorectal adenocarcinoma, were maintained to establish tight junctions during culture. *L*. *johnsonii* MG cells labelled with CFDA were then incubated with Caco-2 cells. After washing with PBS, the bound *L*. *johnsonii* MG cells were monitored using a fluorescent cell imager. When CFDA–MG (+GAPDH) was used, fluorescence was observed on ZO-1-labelled tight junctions; however, the fluorescence completely disappeared when CFDA–MG (−GAPDH) was used (Figure 1B). The number of *L*. *johnsonii* MG cells significantly decreased when CFDA–MG (−GAPDH) was used (Figure 1C). In addition, more than 55% of the *L*. *johnsonii* MG cells bound to Caco-2 cells were localised at tight junctions (Figure 1C). These results strongly suggest that the presence of GAPDH on the cell surface has a critical impact on the binding of *L*. *johnsonii* MG cells to tight junctions in Caco-2 cells.

### 3.2. Interaction of GAPDH with JAM-2

**Figure 2:**
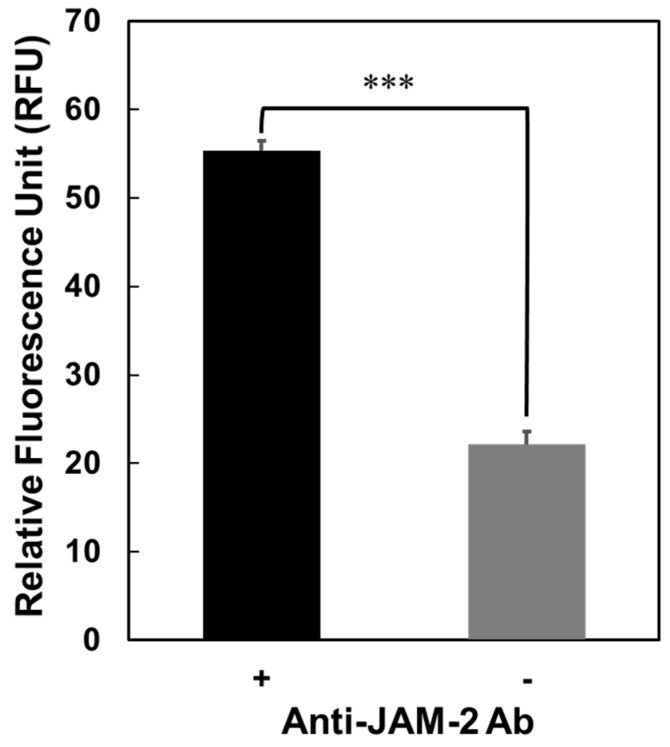
Specific binding of GAPDH to JAM-2. JAM-2 in mouse gut proteins was captured using the anti-JAM-2 antibody coated on a microplate. Cy3-labelled GAPDH was used to assess the interaction. Error bars represent the mean ± SEM (n = 3); *** *p* < 0.001 (one-way ANOVA).

Figure 1 presents the localisation of *L*. *johnsonii* MG at tight junctions in Caco-2 cells. The interaction of GAPDH with JAM-2, a matrix protein in tight junctions, was confirmed in a previous study [19]. JAM-2 was captured on a microplate using an anti-JAM-2 antibody; its interaction with Cy3–GAPDH was then assessed. As shown in Figure 2, Cy3–GAPDH could bind to the JAM-2 captured using the anti-JAM-2 antibody; however, it did not bind to wells that did not contain the anti-JAM-2 antibody. This result suggests that GAPDH, which interacts with JAM-2, plays a crucial role in the binding of *L*. *johnsonii* MG to tight junctions.

### 3.3. Binding of L. johnsonii MG to the Mouse Gut

**Figure 3:**
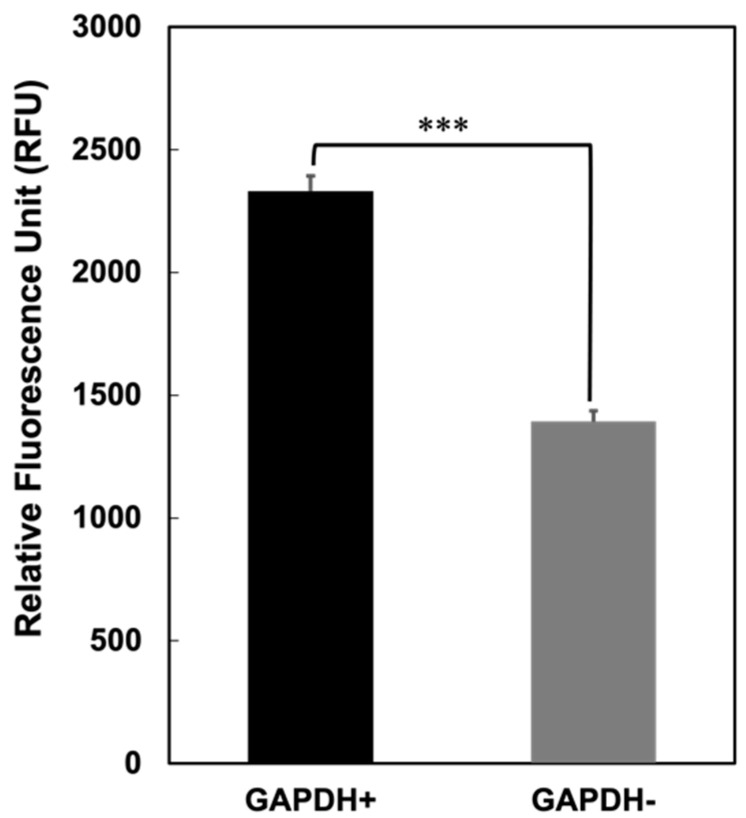
Binding of Cy3-labelled *L. johnsonii* MG with or without GAPDH to the mouse gut. Error bars represent the mean ± SEM (n = 5); *** *p* < 0.001 (one-way ANOVA).

To determine the effect of GAPDH on the adherence of *L*. *johnsonii* MG to the gut, fresh intestines isolated from the BALB/c mice were used to perform binding assays. CFDA-labelled *L*. *johnsonii* MG cells were injected into the fixed mouse intestines and incubated for 30 min at 30 °C. After washing with PBS, the bound bacterial cells were released using 1 M NaCl–PBS. Fluorescence was then quantified using a microplate reader. Significantly higher fluorescence was observed in *L*. *johnsonii* MG cells that displayed GAPDH on their surface than in those that did not display GAPDH (Figure 3). This result suggests that *L*. *johnsonii* MG crucially impacts intestinal binding through the interaction of cell surface GAPDH with gut components, such as JAM-2. 

### 3.4. Function of GAPDH on Tight Junctions

**Figure 4:**
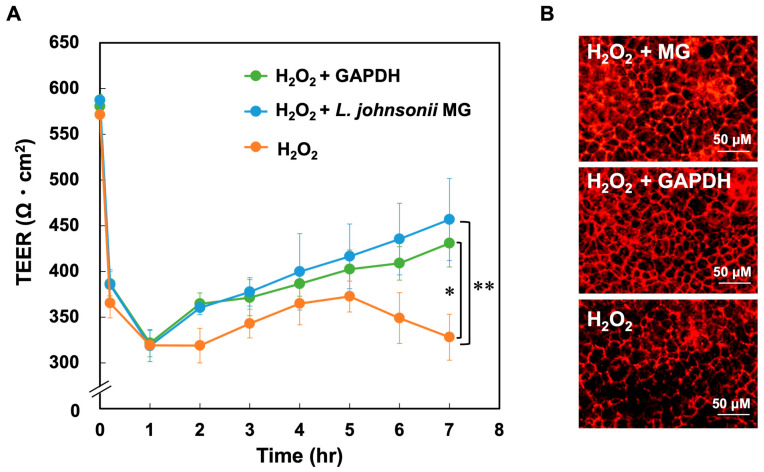
(**A**) Changes in the TEER values of Caco-2 cells during culture after H_2_O_2_ treatment and *L. johnsonii* MG or GAPDH treatment. H_2_O_2_ vs. H_2_O_2_ + GAPDH; *p* = 0.02. H_2_O_2_ vs. H_2_O_2_ + *L. johnsonii* MG; *p* = 0.006. (**B**) Caco-2 cells were stained with an anti-ZO-1 antibody and Cy3-conjugated anti-mouse IgG after H_2_O_2_ treatment and *L. johnsonii* MG or GAPDH treatment. * *p* < 0.05 and ** *p* < 0.01 indicate significant differences between the H_2_O_2_ and H_2_O_2_–GAPDH groups and between the H_2_O_2_ and H_2_O_2_–MG groups, respectively.

Caco-2 cells were exposed to H_2_O_2_ and then treated with purified GAPDH or *L*. *johnsonii* MG to assess the effect of GAPDH on the integrity of gut barrier function. As shown in Figure 4A, the TEER values in all the groups exceeded 550 Ω cm^2^ after 10 days of cultivation and decreased below 320 Ω cm^2^ after 1 h of H_2_O_2_ treatment. The TEER values in the GAPDH-treated and *L. johnsonii* MG-treated groups (H_2_O_2_–GAPDH and H_2_O_2_–MG groups, respectively) after H_2_O_2_ treatment recovered significantly faster than those in the H_2_O_2_ group. The TEER value in the H_2_O_2_–GAPDH group exceeded 430 Ω cm^2^ at 7 h, whereas that in the H_2_O_2_ group was only 350 Ω cm^2^ at 5 h; this value decreased thereafter. Tight junctions were visualised by immunostaining conducted by using an anti-ZO-1 antibody. As shown in Figure 4B, the tight junctions were markedly damaged in the H_2_O_2_ group but were improved in the H_2_O_2_–GAPDH and H_2_O_2_–MG groups. These results suggest that GAPDH without cellular components plays an important role in the repair of damaged tight junctions.

### 3.5. Differentially Expressed Genes in Caco-2 Cells after GAPDH Treatment

**Figure 5:**
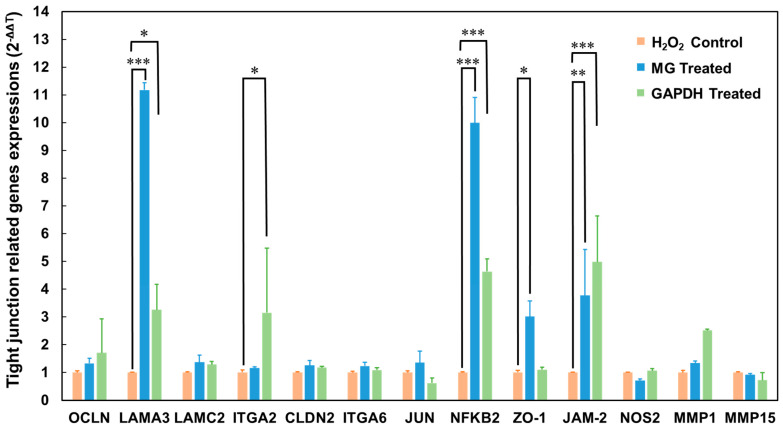
Tight junction-related gene expression analysis in Caco-2 cells after H_2_O_2_ treatment and *L. johnsonii* MG or GAPDH treatment (n = 3). * *p* < 0.05, ** *p* < 0.01 and *** *p* < 0.001 denote significant differences compared with the H_2_O_2_ control.

The interaction of GAPDH with JAM-2 in tight junctions contributed to the anti-inflammatory effect of *L. johnsonii* MG in Caco-2 cells, with the damaged tight junctions in Caco-2 cells displaying a significant recovery after incubation with GAPDH (Figure 4). Therefore, to understand the mechanism underlying the role of GAPDH in the integrity of tight junctions, we assessed the differences in tight-junction-related gene expression levels between the H_2_O_2_ and H_2_O_2_–GAPDH groups observed in a previous study [19]. We quantified the gene expression in Caco-2 cells after the challenge with *L*. *johnsonii* MG and GAPDH in the present study. Based on our previous assessment, we selected various genes involved in transcriptional regulation, the extracellular matrix, apoptosis, and inflammation. Among the selected genes, the levels of LAMA3, ITGA2, NFKB2, and JAM-2 genes were significantly upregulated after incubation with the GAPDH (Figure 5).

### 3.6. Identification of the Region in GAPDH That Binds to the Host Component

**Figure 6:**
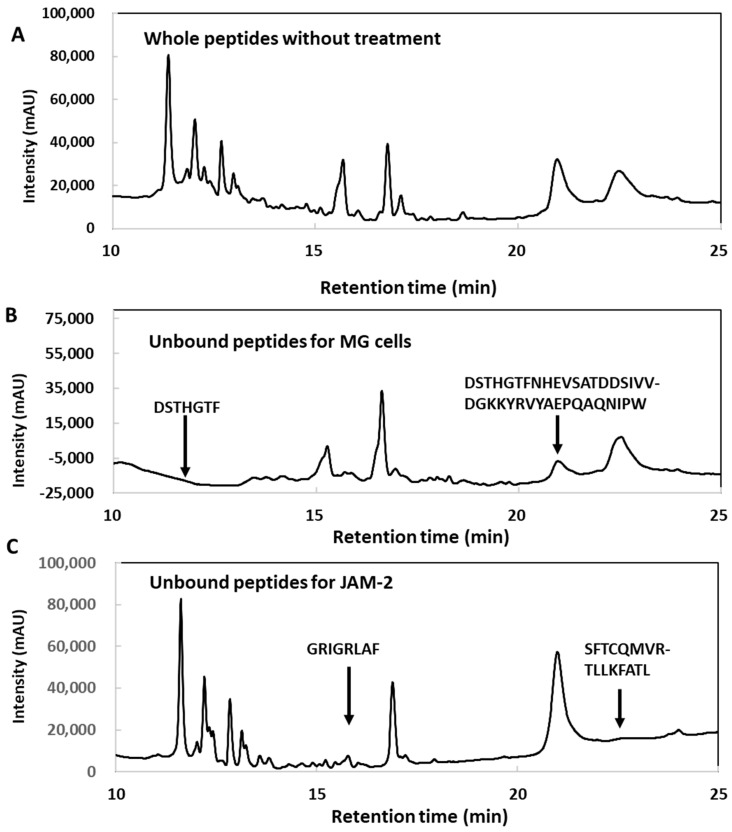
HPLC analysis of peptides released from GAPDH without treatment (**A**) after binding to MG (**B**) and after binding to JAM-2 (**C**).

**Figure 7:**
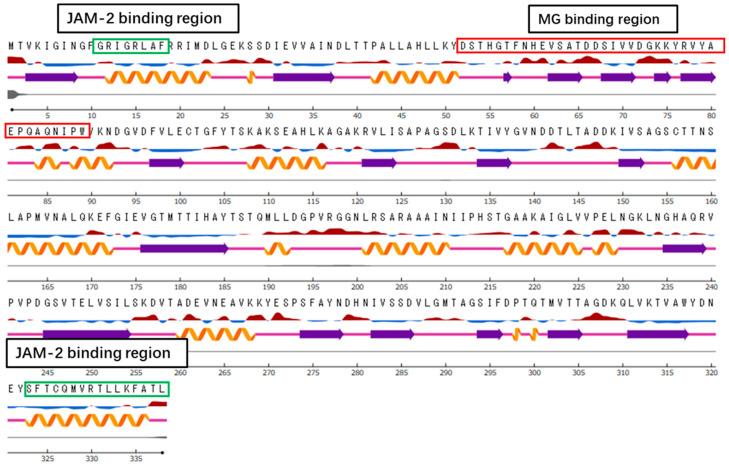
Homology model of surface-associated GAPDH secreted from *L. johnsonii* MG. The NetSurfP (https://services.healthtech.dtu.dk/service.php?NetSurfP-2.0 (accessed on 18 January 2023)) server was used for model building. Predicted peptide regions needed for JAM-2 protein binding and MG cell binding were added.

GAPDH was considered to interact with the *L*. *johnsonii* MG cell surface and JAM-2 was found to localise in tight junctions. Therefore, the specific regions of GAPDH that interact with the *L*. *johnsonii* MG cell surface and JAM-2 were assessed. GAPDH was partially digested with chymotrypsin and the peptide profiles of the hydrolysate were compared using HPLC, with or without incubation, with 5 M LiCl-treated *L*. *johnsonii* MG (Li–MG) and JAM-2. The decreased peaks noted after incubation with Li–MG and JAM-2 were analysed using HPLC. Furthermore, the decreased peaks were collected and the molecular weight was analysed using TOF–MS analysis (Figure 6). Two peaks were reduced after incubation with JAM-2, and two peaks were reduced after incubation with *L*. *johnsonii* MG. Thus, four major peaks were found to decrease in the HPLC analysis and were considered to have affinities for JAM-2 and Li–MG in the binding test (Figure 6). The N-terminal sequence ^11^GRIGRLAF^18^ and the C-terminal sequence ^323^SFTCQMVRTLLKFATL^338^ in GAPDH were predicted to interact with JAM-2 (Figure 7). In contrast, the N-terminal sequence ^52^DSTHGTFNHEVSATDDSIVVDGKKYRVYAEPQAQNIPW^89^ was predicted to be a specific region of the GAPDH that could be integrated into the *L*. *johnsonii* MG cell surface.

### 3.7. Bioinformatics Analysis

An observation of the *L. johnsonii* MG GAPDH sequence against the Protein Data Bank revealed *L. acidophilus* GAPDH (PDB:5J9G) as the closest structural homologue, with 92.90% identity. *L. johnsonii* MG GAPDH lacks a signal peptide sequence, transmembrane domain, and membrane-anchoring domain. The structural difference between the MG-binding site rich in the exposed region and the JAM-2-binding sites rich in the buried regions with helical structures was confirmed in the secondary structure model, predicted using the NetSurfP server (Figure 7). All of the proposed models predicted the helical structure of the C-terminal sequence as a precondition for interaction with the host component.

### 3.8. Docking Analysis of GAPDH Fragments

**Figure 8:**
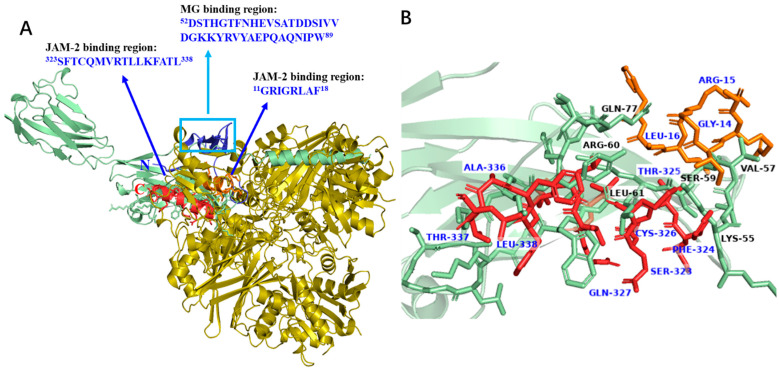
Predicted interactions of GAPDH with JAM-2 in tight junctions and *L. johnsonii* MG cells (**A**). Interacting residues between JAM-2 (black) and GAPDH (blue) (**B**). The 3D structure of GAPDH was constructed using the SWISS-MODEL and the latest version of AlphaFold (https://alphafold.ebi.ac.uk (accessed on 9 March 2023)) was used to predict the 3D structure of the JAM-2 sequence.

**Figure 9:**
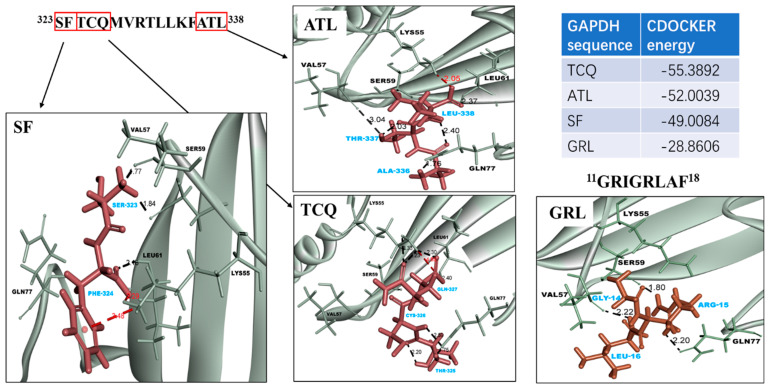
Docking simulation of GAPDH fragments and JAM-2 protein. Molecular docking simulations were performed using the CDOCKER program in Discovery Studio (DS) 2018 software. Comparison of different GAPDH fragments in the docking simulation. Interaction energies were calculated after docking. Molecular docking results showing the interacting residues between SF, TCQ, and ATL (all blue) in ^323^SFTCQMVRTLLKFATL^338^ and GRL (blue) in ^11^GRIGRLAF^18^ and JAM-2 (black). The bound hydrogen is shown in black, and the electrostatic interaction is shown in red. The CHARMM force field base docking tool was used in CDOCKER for performing molecular dynamics module calculations to predict the putative peptide–protein complexes.

Based on our results, we predicted a possible interaction mechanism of GAPDH with JAM-2 and *L*. *johnsonii* MG cells. An assessment of the binding region revealed that the N-terminal sequence ^11^GRIGRLAF^18^ and the C-terminal sequence ^323^SFTCQMVRTLLKFATL^338^ in the GAPDH were important regions for JAM-2 binding. To understand the interactions between the selected peptides and JAM-2, we performed a docking analysis based on the predicted 3D structure of JAM-2. The small peptide sequences located in the selected regions displayed good binding to the adjacent regions of JAM-2, ranging from 50 to 80 aa positions in the sequence (Figure 8A). Specifically, LYS^55^, VAL^57^, SER^59^, ARG^60^, LEU^61^, and GLN^77^ of JAM-2 interacted with GAPDH (Figure 8B). These amino acids in JAM-2 exhibited a hydrogen bonding interaction with specific amino acids in the GAPDH (JAM-2–GAPDH), namely LYS^55^–GLN^327^ (2.30 Å), VAL^57^–THR^337^ (3.04 Å), VAL^57^–SER^323^ (1.77 Å), VAL^57^–LEU^16^ (2.22 Å), SER^59^–SER^323^ (1.84 Å), SER^59^–ARG^15^ (1.80 Å), LEU^61^–GLN^327^ (2.04 Å), LEU^61^–LEU^338^ (2.37 Å), LEU^61^–PHE^324^ (2.16 Å), GLN^77^–CYS^326^ (2.59 Å), GLN^77^–THR^325^ (1.75 Å), GLN^77^–ALA^336^ (1.76 Å), and GLN^77^–ARG^15^ (2.20 Å) (Figure 8B). Meanwhile, the binding of JAM-2 to the GAPDH was influenced by electrostatic interactions, e.g., LYS^55^–GLN^317^ (4.85 Å), LYS^55^–LEU^338^ (2.05 Å), and LYS^55^–PHE^324^ (3.48 Å) (Figure 9) The CDOCKER energies of the selected peptide sequence were compared. All peptides located at the C-terminus had lower energy, suggesting that the helical structure at the C-terminus of GAPDH influences binding to JAM-2 (Figure 9). 

## 4. Discussion

Our previous study revealed the anti-inflammatory effect of *L. johnsonii* MG based on its interaction with JAM-2 at tight junctions [19]. GAPDH plays an important role in the interactions between *L*. *johnsonii* MG and tight junctions in Caco-2 cells. In the present study, H_2_O_2_-damaged tight junctions in Caco-2 cells were rescued via treatment with purified GAPDH, as monitored based on TEER values. The biological impact on tight junctions was similar to that of *L*. *johnsonii* MG-coating GAPDH (Figure 4). Moreover, the host responses involved in transcriptional regulation, extracellular matrix formation, apoptosis, and inflammation observed after treatment with GAPDH in the present study (Figure 5) were similar to those observed in a previous study using *L*. *johnsonii* MG [19]. These results strongly suggest that GAPDH enhances the integrity of tight junctions by interacting with JAM-2 in Caco-2 cells, as reported when previously using *L*. *johnsonii* MG [19]. 

GAPDH exerts a postbiotic effect, which is a concept focused on heat-killed probiotic effects and metabolites released from bacteria, including bacteriocins and antibacterial proteins, exopolysaccharide (EPS), and lipoteichoic acid (LTA) [23]. Various bacteriocins originating from lactic acid bacteria have been reported and reviewed [24]. Various types of bacteriocins and antibacterial proteins exhibited effects on pathogens, such as *Staphylococcus aureus*, *Pseudomonas fluorescens*, *P. aeruginosa*, *Salmonella typhi*, *Shigella flexneri*, *Listeria monocytogenes*, *Escherichia coli* O157:H7, and *Clostridium botulinum*. EPS produced by *L. gasseri* [25] and *L. kefiranofaciens* DN1 [26] exhibited in vitro antibacterial activity against several pathogens. Electron microscopic analysis suggested that the disruption of the cell membranes of gram-positive and gram-negative pathogenic bacteria by EPS was the underlying mechanism [27]. LTA produced by *Bifidobacterium animalis* subsp. *lactis* BPL1 exhibited fat-reducing properties [28]. Moreover, peptide B7, a bioactive peptide secreted by the probiotic bacterium *B. longum*, showed a trend of reducing the secretion of the proinflammatory cytokine IFN-γ and increasing the secretion of the chemoattractant CCL-2 [29].

GAPDH is a ubiquitous enzyme involved in glycolysis. It has been reported to be a member of the moonlighting protein family, which has multiple intracellular functions [17,30,31,32]. GAPDH secreted on bacterial surfaces interacts with mucin [17] and cell matrix components [33,34]. In the mouse intestine, only a small percentage of bacteria (0.5%) can secrete GAPDH on their surfaces [19]. GAPDH exhibits multiple adhesion abilities to intestinal epithelial components, such as mucin, fibronectin, and JAM-2 [15,16,17,18,19]. However, little is known about its specific binding to different types of epithelial components. In the present study, we identified specific peptides with an affinity for JAM-2 by detecting decreased peaks after incubation with GAPDH hydrolysate and JAM-2 (Figure 6). As a result, we used GAPDH digested with chymotrypsin for the JAM-2 binding assay. Moreover, we identified ^11^GRIGRLAF^18^ in the N-terminal sequence and ^323^SFTCQMVRTLLKFATL^338^ in the C-terminal sequence as peptides binding to JAM-2 (Figure 6 and Figure 7). The N-terminus may be separated from the C-terminus; however, both termini were found to be located adjacent to the tetrameric 3D structure in the docking analysis (Figure 8) and in the predicted 3D structure of GAPDH [35]. Both peptides released from the N- and C-terminal sequences interacted with the JAM-2 protein in a limited area, ^55^KTVSSRLEWKKLGRSVSFVYYQQ^77^, in JAM-2. This result suggests that GAPDH with a tetrameric 3D structure is essential for interacting with JAM-2 and enhancing the integrity of tight junctions. This also suggests that GAPDH located on *L. johnsonii* MG has a tetrameric structure to interact with JAM-2 and exhibit similar biological effects on tight junctions in Caco-2 cells. *L. reuteri* ZJ617 possessing more surface GAPDH exhibited greater cell membrane permeability and adhesive activity towards mucin; higher adhesion to the jejunum, ileum, and colon of piglets; and a lower incidence rate of diarrhoea in piglets compared with *L. reuteri* ZJ617 possessing less surface GAPDH [36]. A structural analysis suggested that there were interactions of GAPDH with glycan (GalNAc 1-O-Ser, GalNAc Ser, and Gal GalNAc) in mucin through the formation of hydrogen bonds with Met^1^, Ser^2^, Val^3^, Ser^29^, Asp^30^, and Ile^31^ in GAPDH. The tetrameric structure of GAPDH from *L. acidophilus* also exhibited mucin-binding and haemagglutination activities, indicating a carbohydrate-like binding mechanism [37]. Interactions of GAPDH with mannose, galactose, N-acetylgalactosamine, and N-acetylglucosamine were suggested to have taken place. These predicted mucin-binding peptides differed from the peptides needed for JAM-2 binding, suggesting that the different interactions of GAPDH with JAM-2 and mucin are important for multifunctional roles. 

GAPDH has been detected as a protein on the bacterial cell surface or as a protein secreted by various bacteria, such as *S. aureus* [38], *Streptococcus agalactiae* [39], *L. plantarum* [17], and *L. johnsonii* [19]. Hence, GAPDH can interact with proteins of the host organism but lacks any known motif for the C-terminal hydrophobic tail. In the peptide binding assay, ^52^DSTHGTFNHEVSATDDSIVVDGKKYRVYAEPQAQNIPW^89^ with a pI value of 4.86 was predicted to be the specific sequence required for interaction with the *L*. *johnsonii* MG cell surface. The reasons for GAPDH secretion into the medium, without a signal sequence and localisation, on the cell surface of various bacterial species remain unclear; however, the peptide fragment specified in the present study may be useful for further detailed studies on this interaction.

In the present study, bacterial cellular components were not necessary for the enhancement of tight junctions. Therefore, GAPDH can be used as a postbiotic *Lactobacillus* component. In industrial applications, postbiotics have some advantages over probiotics because of their ease of processing, stability, and storage [40]. Recently, the use of postbiotic mixtures in functional foods [41,42], nutrition [43] and allergy treatment [44] has been reviewed; however, there is still a gap in the literature regarding the steps before the use of postbiotics in food. Among the different types of milk fermented with various lactic acid bacteria, anti-hypertensive peptides observed in *Lactobacillus*-fermented milk [45] were applied to many kinds of functional foods after confirming their effects in animal and clinical studies [46,47,48] and developing an enzymatic method [49]. Thus, GAPDH has great potential as a postbiotic active component in functional foods if some preparative methods are developed. However, a quantification of the GAPDH in faecal samples should be performed, if it is to be used as a functional food material, in order to determine the appropriate dosage and consider its in vivo effect.

## 5. Conclusions

GAPDH, a moonlighting protein released from *L. johnsonii* MG, was found to induce tight junction-related gene expression by interacting with JAM-2, enhance tight junctions, and rescure H_2_O_2_-treated damaged tight junctions in Caco-2 cells. Two short peptide regions, ^11^GRIGRLAF^18^ at the N-terminus and ^323^SFTCQMVRTLLKFATL^338^ at the C-terminus, interacted with the adjacent regions of JAM-2. The long peptide ^52^DSTHGTFNHEVSATDDSIVVDGKKYRVYAEPQAQNIPW^89^ interacted with the surface of *L*. *johnsonii* MG cells. These results suggest a novel moonlighting function of GAPDH through the interaction of specific regions with JAM-2.

## Data Availability

The data presented in this study are available on request from the corresponding author.

## Supplementary Materials

The following supporting information can be downloaded at https://www.mdpi.com/article/10.3390/microorganisms11061393/s1.
