# Supplementary figures and images for: GAPDH Released from Lactobacillus johnsonii MG Enhances Barrier Function by Upregulating Genes Associated with Tight Junctions
Source: Microorganisms. 2023 May 25;11(6):1393. doi: 10.3390/microorganisms11061393 (PMC10302070; doi:10.3390/microorganisms11061393)

## Slide 1
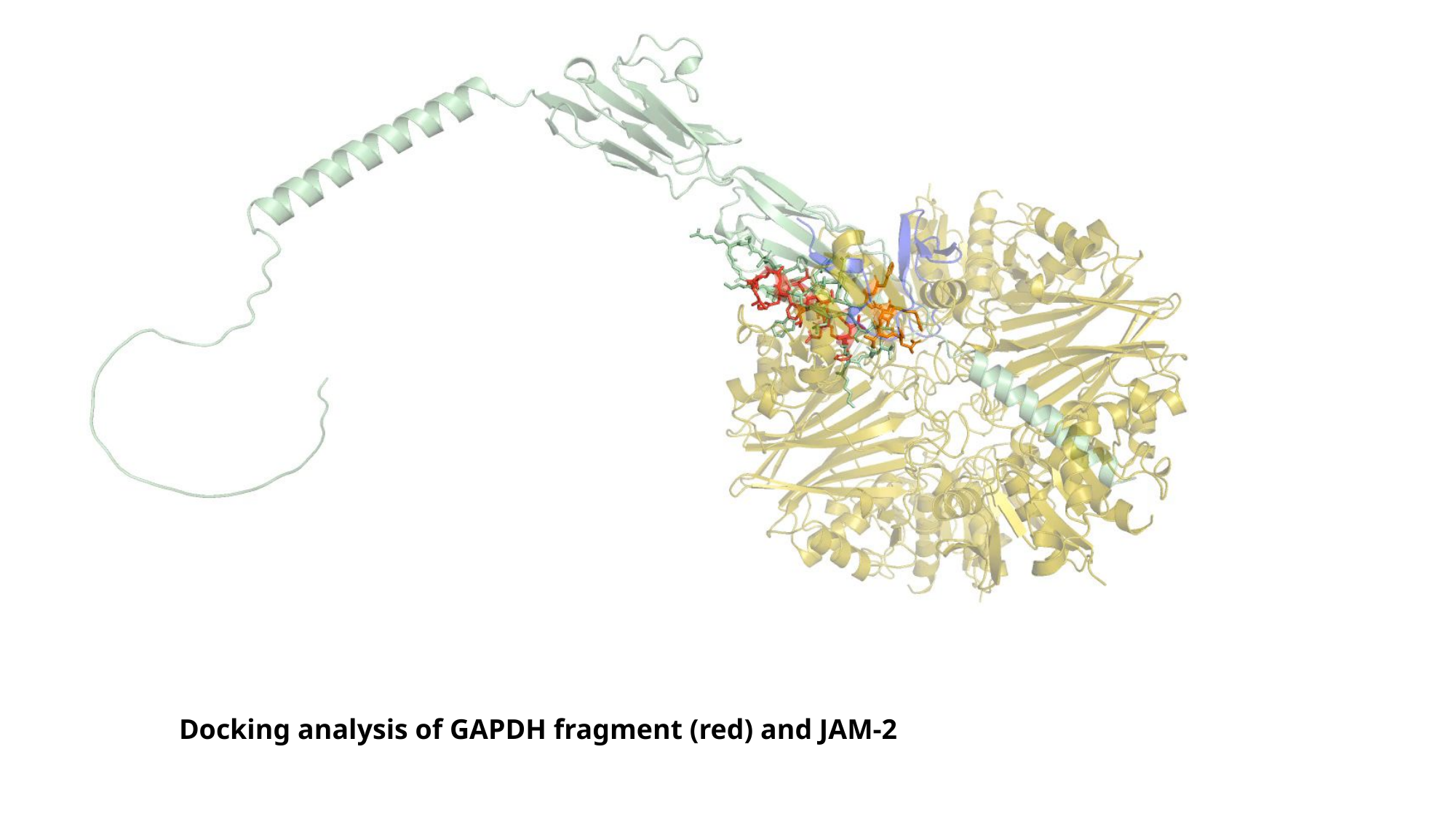

Docking analysis of GAPDH fragment (red) and JAM-2

Supplement: Supplementary file 1 [file microorganisms-11-01393-s001.zip › Docking movie.pptx]
